# Nutritional Value and Productivity Potential of the Marine Microalgae *Nitzschia laevis*, *Phaeodactylum tricornutum* and *Isochrysis galbana*

**DOI:** 10.3390/md22090386

**Published:** 2024-08-27

**Authors:** Xue Lu, Shufang Yang, Yongjin He, Weixuan Zhao, Man Nie, Han Sun

**Affiliations:** 1 Institute of New Materials and Advanced Manufacturing, Beijing Academy of Science and Technology, Beijing 100089, China; luxue@bjast.ac.cn (X.L.); zhaoweixuan@bjast.ac.cn (W.Z.); nieman@bjast.ac.cn (M.N.); 2 College of Chemistry and Environmental Engineering, Shenzhen University, Shenzhen 518071, China; shufang-yang@szu.edu.cn; 3 College of Life Science, Fujian Normal University, Fuzhou 350117, China; yongjinhe@fjnu.edu.cn; 4 Engineering Research Center of Watershed Carbon Neutrality of Ministry of Education, Center for Algae Innovation & Engineering Research, School of Resources and Environment, Nanchang University, Nanchang 330031, China

**Keywords:** fucoxanthin, polyunsaturated fatty acids, *Nitzschia laevis*, *Phaeodactylum tricornutum*, *Isochrysis galbana*

## Abstract

Microalgae are considered promising sustainable feedstocks for the production of food, food additives, feeds, chemicals and various high-value products. Marine microalgae *Phaeodactylum tricornutum*, *Isochrysis galbana* and *Nitzschia laevis* are rich in fucoxanthin, which is effective for weight loss and metabolic diseases. The selection of microalgae species with outstanding nutritional profiles is fundamental for novel foods development, and the nutritional value of *P. tricornutum*, *I. galbana* and *N. laevis* are not yet fully understood. Hence, this study investigates and analyzes the nutritional components of the microalgae by chromatography and mass spectrometry, to explore their nutritional and industrial application potential. The results indicate that the three microalgae possess high nutritional value. Among them, *P. tricornutum* shows significantly higher levels of proteins (43.29%) and amino acids, while *I. galbana* has the highest content of carbohydrates (25.40%) and lipids (10.95%). Notwithstanding that *P. tricornutum* and *I. galbana* have higher fucoxanthin contents, *N. laevis* achieves the highest fucoxanthin productivity (6.21 mg/L/day) and polyunsaturated fatty acids (PUFAs) productivity (26.13 mg/L/day) because of the competitive cell density (2.89 g/L) and the advantageous specific growth rate (0.42/day). Thus, compared with *P. tricornutum* and *I. galbana*, *N. laevis* is a more promising candidate for co-production of fucoxanthin and PUFAs.

## 1. Introduction

Microalgae fix CO_2_ through the Calvin cycle to convert it into valuable organic compounds through various intracellular metabolic pathways, such as pigments, proteins, polysaccharides and lipids [1,2,3,4]. Many of these compounds, including carotenoids, polyunsaturated fatty acids (PUFAs), bioactive poly/monosaccharides, proteins and peptides containing essential amino acids (EAAs), present high nutritional value and health-beneficial functions, such as anti-oxidation, anti-inflammatory, anti-cancer, anti-obesity and anti-diabetes activities [4]. Accordingly, microalgae are promising to take the place of traditional food sources as new food and functional food products, and due to their well-rounded chemical composition, they can be used to enhance the nutritional value of foods. For example, adding *Spirulina* in bread improved its nutritional quality, increasing protein content and certain essential amino acids (threonine, methionine, isoleucine and leucine) by 39.04% compared to untreated bread [5]. Incorporating 5.41% *Spirulina* significantly enhanced the nutritional value of donuts, with protein content rising from 7.37% to 12.19% and lipid content increasing from 5.82% to 12.11% [6]. Moreover, marine microalgae, in particular, offer unique bioactive compounds due to their ability to thrive in diverse marine environments [7]. Hence, it is essential to acquire adequate knowledge about the biochemical composition of marine microalgal species in order to exploit their potential in different fields. 

Among the typically bioactive components in microalgae, proteins and polysaccharides constitute a significant proportion of the dry cell weight (DCW). However, the types and amounts of nutrients vary greatly among different microalgae species. For instance, the protein content of *Chlorella* sp. is around 40–50% of the DCW, whereas *Spirulina* sp. has a protein content as high as 70% [8]. The chrysolaminarin content of *Odontella aurita* is notably high [9,10], constituting important nutritional components of its cellular sugars, while some microalgae have higher levels of fatty acids instead [11,12,13,14]. The nutritional value of microalgae depends not only on the nutrients content in cells but also on the proportions and composition of each nutrient component. For example, the level of EAAs within proteins determines their nutritional value, and the content of essential PUFAs, such as arachidonic acid (ARA), eicosapentaenoic acid (EPA) and docosahexaenoic acid (DHA) in fatty acids reflects the nutritional value of algal oil. Among the fatty acids, PUFAs have more than one double bond in their carbon chain structure, and they are considered as high-value compounds in health food industry. There are two classes of PUFAs, *n*-6 and *n*-3, which are synthesized from linoleic acid and linolenic acid, respectively. In addition, alpha-linolenic acid, EPA and DHA are the key *n*-3 fatty acids. DHA and EPA are nutritionally significant PUFAs produced in significant amounts by some marine microalgae, and they have been paid particular interest due to their bioactivities for human fitness [6,15]. It was reported that regular consumption of EPA and DHA supplements could reduce inflammation and prevent cardiovascular disease [16,17,18,19]. The production of microalgal PUFAs has become more cost-effective compared to the production of biofuel, and in recent years, a large number of producers have shifted their focus towards PUFAs production.

Apart from these common nutrients, species like *Phaeodactylum tricornutum*, *Isochrysis galbana*, *Nitzschia laevis* and *O. aurita* are rich in fucoxanthin [20,21,22], which cannot be synthesized artificially. It is one of the primary carotenoids in marine brown seaweeds, diatoms and golden algae [23,24]. Recent studies have focused on synthesizing fucoxanthin using *P. tricornutum* and *I. galbana*, given its potential for beneficial activities, including anti-oxidation and anti-obesity effects [24,25,26,27]. Furthermore, the selection of industrial microalgae species with outstanding nutritional profiles is fundamental for the successful development of novel foods. Hence, a detailed analysis and evaluation of the nutritional value of the microalgae is essential for the selection of the suitable microalgae for specific food technology applications, consequently facilitating the food industry. Therefore, this study selects three marine microalgae species (*P. tricornutum*, *I. galbana* and *N. laevis*) rich in fucoxanthin and PUFAs to investigate and analyze their growth characteristics and main nutritional components, thereby exploring their potential in the industrial production of high-value products.

## 2. Results and Discussion

### 2.1. Growth Characteristics and Carbon Partitioning of P. tricornutum, I. galbana and N. laevis

**Figure 1:**
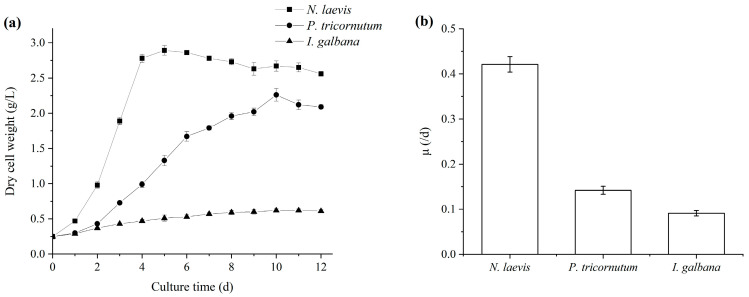
Growth curves (**a**) and maximum specific growth rates (**b**) of *N. laevis*, *P. tricornutum* and *I. galbana* under mixotrophic cultivation. Values are mean ± SD of at least three independent experiments.

The industrialization potential of microalgae mainly depended on the productivities of high-value products [28,29,30], which were closely related with their growth characteristics and the biosynthesis of target products [31,32]. Among the three microalgae, *N. laevis* UTEX 2047 was able to grow under mixotrophic cultivation, heterotrophic cultivation and autotrophic cultivation, but the biomass concentration in autotrophic cultivation was rare low. During the cultivation process, the addition of light usually incurred significantly higher costs. Thus, considering the production cost, heterotrophic cultivation was chosen for *N. laevis* UTEX 2047 in this study. In terms of cell growth, *N. laevis* UTEX 2047 exhibited a notably higher specific growth rate compared to *P. tricornutum* UTEX 646 and *I. galbana* UTEX 2307, as shown in Figure 1, indicating faster proliferation to achieve higher final cell densities. Studies revealed that both *P. tricornutum* and *I. galbana* required light for growth and struggled to achieve high biomass concentrations [33,34,35], limiting their development and utilization to some extent. Thus, mixotrophic cultivation was chosen for *P. tricornutum* and *I. galbana* due to the ability to achieve higher cell densities with the addition of organic substrates compared with autotrophic cultivation. Previous research utilizing flat-panel photobioreactors achieved maximum biomass concentrations of only 4.10 g/L and 3.99 g/L for *P. tricornutum* and *I. galbana*, respectively [36,37]. In contrast, *N. laevis* could be cultured under both light and dark conditions, with high glucose utilization rates facilitating the attainment of high biomass concentrations [38,39]. Early studies by Wen et al. demonstrated that the high cell density of 40 g/L could be achieved by using perfusion cultivation methods for *N. laevis* [40].

Biochemical composition of microalgae cells that included proteins, carbohydrates, lipids and pigments could provide an indication of their nutritional value. The intracellular composition of total proteins, carbohydrates, lipids and pigments in three microalgae are summarized in Table 1. It was observed that *P. tricornutum* UTEX 646 exhibited the highest protein content at 43.29% of the DCW, which was significantly higher than that of the other two species. *I. galbana* showed the highest total carbohydrate content at 25.40%, followed by *N. laevis* at 21.97% and *P. tricornutum* at 14.85%. All three microalgae were rich in lipids, with the highest lipid content found in *I. galbana,* followed by *P. tricornutum* and *N. laevis*. As for pigment content, *P. tricornutum* showed significantly higher levels compared to *I. galbana* and *N. laevis*, exceeding their pigment contents by two-fold and three-fold, respectively. The nutritional value of the proteins, polysaccharides and lipids mainly depended on amino acid composition, monosaccharide composition and the presence of PUFAs. 

### 2.2. Amino Acid Composition of P. tricornutum, I. galbana and N. laevis

The total protein content of microalgae varied due to different species and up to 70% of the DCW [8,41,42,43]. The amino acid profile, including the content, proportion and availability of amino acids, was an important parameter of the protein quality and nutritional value [44]. The amino acid profiles of *P. tricornutum*, *I. galbana* and *N. laevis* were compiled in Table 2, and the Food and Agriculture Organization of the United Nations (FAO/WHO) proposed a reference standard with the recommended content of the EAAs in a protein or a mixture of proteins.

As shown in Table 2, the amino acid profiles of *P. tricornutum*, *I. galbana* and *N. laevis* were deficient for tryptophan. It was evident that *P. tricornutum* exhibited higher concentrations of most amino acids compared to the others, consistent with its highest protein content as shown in Table 1. Among non-essential amino acids (NEAA), alanine, serine, aspartic acid, glutamic acid and arginine were presented in notable amounts, exceeding 1% of the DCW in all three microalgae. EAAs, except for methionine, which is below 0.5% of the DCW, were generally presented in higher concentrations, particularly leucine, which exceeds 2% of the DCW in both *P. tricornutum* and *I. galbana*. Additionally, histidine, which was unable to be synthesized by infants, was sometimes considered essential. All three microalgae contained varying amounts of histidine, with similar and relatively high levels observed in *P. tricornutum* and *I. galbana*. However, the lysine in three marine microalgae were lower than those reported in *Chlorella vulgaris*, *Arthrospira maxima* and *Arthrospira platensis*, which might suggest freshwater microalgae are more suitable as feed because lysine was typically the first limiting amino acid in aquaculture [44]. The lack or low content of tryptophan might be due to the high-salinity environment being unfavorable for its biosynthesis. The total content of EAAs was notably higher in *P. tricornutum* (9.00%) and *I. galbana* (8.17%) compared to *N. laevis*. Therefore, considering the protein nutrition, *P. tricornutum* UTEX 646 and *I. galbana* UTEX 2307 demonstrated advantages over *N. laevis* UTEX 2047 among the three microalgae.

According to the ideal protein model proposed by FAO/WHO standard, the ratio of EAAs to total amino acids (EAA/TAA) of good quality was about 40%, and the ratio of essential to non-essential amino acids (EAA/NEAA) was more than 60% [45]. As presented in Table 2, *P. tricornutum* and *I. galbana* showed better qualities compared with *N. laevis*. The EAA indices reflected the average supply of EAAs in food proteins. The higher EAA index (EAAI) value of food protein is to 100, the higher the nutritional value. Hence, the protein of *P. tricornutum* presented more valuable than that of the other two microalgae. According to Table 2, *N. laevis* UTEX 2047 may not be the best choice to obtain protein for food and feed application.

### 2.3. Monosaccharide Profiles of P. tricornutum, I. galbana and N. laevis

Compared to other sources, polysaccharides derived from microalgae were reported as stable, safe, versatile, biocompatible and biodegradable [46]. They presented complex biochemical structures according to each microalgal species. In addition, they exhibited beneficial biological characteristics that included anti-oxidant, anti-inflammatory, anti-tumor and anti-microbial activities [47]. Microalgal polysaccharides were predominantly composed of pentose and hexose monosaccharide subunits with many glycosidic bonds [46].

The monosaccharide profiles of three microalgae after polysaccharide hydrolysis are shown in Table 3. It was observed that glucose was the predominant monosaccharide among the 14 identified monosaccharides and uronic acids in all three microalgae. In addition to glucose, mannose content was also notably high in *P. tricornutum* and *N. laevis*, exceeding 40 mg/g. Although *I. galbana* exhibited relatively high proportions of glucose and mannose in its own cellular polysaccharides, these amounts were several times lower compared to those exhibited by *P. tricornutum* and *N. laevis*. This discrepancy could be attributed to the lack of a cell wall in *I. galbana*, resulting in lower polysaccharide content compared to *P. tricornutum* and *N. laevis*. However, *I. galbana* contained comparable or higher levels of galacturonic acid, ribose, galactose, xylose, arabinose and fucose compared to the other two species, and especially notable high levels of arabinose (7.01 mg/g) and fucose (3.39 mg/g) which were significantly higher than those in *P. tricornutum* and *N. laevis*. Galacturonic acid and galactose content were more than twice as high in *I. galbana* compared to *N. laevis*.

*N. laevis* exhibited the highest levels of glucose (529.10 mg/g), mannose (44.26 mg/g), ribose (4.25 mg/g), glucosamine (2.25 mg/g) and galacturonic acid (2.85 mg/g), which were easily to be digested and absorbed deep. Studies indicated that microalgal cell walls consisted of heteropolysaccharides or multiple types of polysaccharides, with glucose, mannose, xylose and galactose typically being the most abundant monosaccharides [47,48,49]. For instance, in *C. vulgaris*, glucogalactan was prevalent in the cell wall polysaccharides, making glucose and galactose the most abundant monosaccharides [50]. The ratio of galacturonic acid to glucuronic acid in cells depended significantly on the microalgae species. While *P. tricornutum* and *N. laevis* had higher total monosaccharide content compared to *I. galbana*, *I. galbana* exhibited advantages in specific monosaccharides such as arabinose and fucose.

### 2.4. Fatty Acids Profiles of P. tricornutum, I. galbana and N. laevis

The characteristic of microalgae to store lipids was extremely valuable to meet the growing demand for the production of food, feed and biofuel. According to the analysis, lipids were the most frequently extracted compounds from microalgae and had the greatest potential for commercialization [11,14,15,51]. The diverse lipid composition of microalgae opens up broad applications, such as biofuel production, animal husbandry/aquaculture feeds, food products and food additives [52].

In this study, all three microalgae were rich in lipids, with *P. tricornutum* and *N. laevis* containing high levels of EPA, as shown in Table 4, while *I. galbana* was abundant in DHA and stearidonic acid (SDA). Linoleic acid and gamma-linolenic acid were essential fatty acids in humans and precursors for the synthesis of ARA, EPA and DHA, the physiological functions of which had been well-known in the medical and health industries, and many PUFAs had already been put into production and utilization. Among the three microalgae, *I. galbana* contained the highest level of C18 unsaturated fatty acid, with C18:1 at 3.17%, C18:3 at 1.05% and C18:4 at 1.20% (DCW), respectively. In addition, the C14:0 content of 1.71% and C16:0 content of 1.82% of *I. galbana* were higher than the other two microalgae. Differently, *P. tricornutum* and *N. laevis* presented high contents of C16:0, C16:1 and C20:5, and the EPA contents even reached over 2% of the DCW, making them the highest natural sources of EPA, and valuable for EPA industrial applications. Overall, the PUFAs within the cells of these three microalgae possessed significant nutritional value.

### 2.5. Fucoxanthin and PUFAs Productivities of P. tricornutum, I. galbana and N. laevis

**Figure 2:**
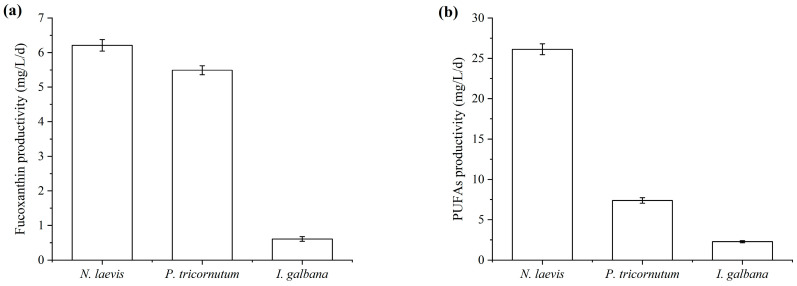
Fucoxanthin productivities (**a**) and PUFAs productivities (**b**) of *N. laevis*, *P. tricornutum* and *I. galbana*. Values are mean ± SD of at least three independent experiments.

Fucoxanthin-rich microalgae, including *P. tricornutum*, *I. galbana*, *Odontella aurita* and *Mallomonas* sp., have been reported to have high fucoxanthin content (1.65% to 2.66%), but their fucoxanthin productivities were low, with the maximum productivity ranging from 1.75 to 7.96 mg/L/day [20,28,29,30,53,54,55]. Industrial-scale fermentation of microalgae for producing high-value products necessitated considerations beyond the intracellular content of the target product, including biomass concentration and the fermentation cost. Especially, despite higher fucoxanthin content in *I. galbana* cells, its slow growth and low biomass concentration resulted in lower fucoxanthin productivity. Although *N. laevis* had the lowest fucoxanthin content among the three microalgae, its rapid growth and ability to achieve high biomass concentrations contributed to the highest fucoxanthin productivity. As shown in Figure 2, the fucoxanthin content of *N. laevis* (0.89%, DCW) was lower than that of *P. tricornutum* (2.43%, DCW) and *I. galbana* (0.98%, DCW), but its fucoxanthin productivity (6.21 mg/L/day) was significantly higher compared to *P. tricornutum* and *I. galbana*. Besides the fucoxanthin, the PUFAs produced by microalgae were also worth to be co-produced for the high value utilization.

Due to the similar physicochemical properties of some microalgal metabolites (such as fatty acids and carotenoids), including molecular weight, polarity, solubility and hydrophobicity, multiple compounds could be extracted from microalgal cells simultaneously. Therefore, many efforts had been devoted to co-production of high-value metabolites by microalgae. For example, *Synechococcus nidulans* and *Spirulina* sp. were used to co-produce phycobiliprotein and carbonic anhydrase [56]; *Dunaliella salina* was selected as a candidate producer of carotenoid and protein [57]. In view of the great commercial application prospect of fucoxanthin and PUFAs, several marine microalgae were explored for the co-production potential [58]. It was reported that *N. laevis* and *P. tricornutum* were promising producers for EPA [36,38,40,54], and *I. galbana* was abundant in DHA and SDA. However, the production process was still faced with many problems such as high production costs and low productivities. As shown in Table 4 and Figure 2, although there were no large differences between the PUFAs contents of *P. tricornutum*, *I. galbana* and *N. laevis*, the PUFAs productivity of *N. laevis* (26.13 mg/L/day) was much higher than that of *P. tricornutum* and *I. galbana.* Given the advantage in fucoxanthin and PUFAs productivities, *N. laevis* was expected to be more attractive for industrial applications compared to the other two microalgae. Nevertheless, each microalgae species has its own benefits, such as the differential production of EPA (*P. tricornutum*), DHA (*I. galbana*) and both (*N. laevis*) omega-3 PUFA species, which command high interest in the food and feed sector.

## 3. Materials and Methods

### 3.1. Strains and Mediums

The *Nitzschia laevis* UTEX 2047, *Phaeodactylum tricornutum* UTEX 646 and *Isochrysis galbana* UTEX 2307 were purchased from the Culture Collection of Algae at the University of Texas-Austin, USA. *N. laevis* was cultured in the modified LDM medium; *P. tricornutum* and *I. galbana* were cultured in the modified f/2 medium.

Modified LDM medium (per L) comprised 892 mL artificial seawater, tryptone 1 g, NaNO_3_ 1 g, Na_2_SiO_3_·9H_2_O 120 mg, K_2_HPO_4_ 7.5 mg, KH_2_PO_4_ 17.5 mg, MgSO_4_·7H_2_O 7.5 mg, Na_2_EDTA 4.5 mg, CaCl_2_·2H_2_O 2.5 mg, FeCl_3_·6H_2_O 0.582 mg, ZnCl_2_ 0.03 mg, CoCl_2_·6H_2_O 0.012 mg, Na_2_MoO_4_·2H_2_O 0.024 mg, MnCl_2_·4H_2_O 0.246 mg, biotin 0.025 mg and VB_12_ 0.015 mg. The medium was adjusted to pH 8.5.

Modified f/2 medium (per L) comprised NaNO_3_ 300 mg, NaH_2_PO_4_·H_2_O 20 mg, Na_2_EDTA·2H_2_O 4.36 mg, FeCl_3_·6H_2_O 3.15 mg, MnCl_2_·4H_2_O 0.18 mg, CoCl_2_·6H_2_O 0.01 mg, ZnSO_4_·7H_2_O 22 μg, CuSO_4_·5H_2_O 9.8 μg, Na_2_MoO_4_·2H_2_O 6.3 μg, thiamin·HCl 100 μg, biotin 0.5 μg and cyanocobalamin 0.5 μg. The culture medium of *P. tricornutum* contain sea salt 20 g/L, Na_2_SiO_3_·9H_2_O 30 mg/L, and the medium was adjusted to pH 8.5. The culture medium of *I. galbana* contain sea salt (Sigma Co., Kawasaki, Japan) 20 g/L, and the medium was adjusted to pH 8.2.

Artificial sea water (per L) comprised NaCl 18 g, MgSO_4_·7H_2_O 2.44 g, KCl 0.6 g, NaNO_3_ 1 g, CaCl_2_·2H_2_O 0.3 g, KH_2_PO_4_ 0.05 g, Tris buffer (Sigma Co.) 1 g, NH_4_Cl 0.0267 g, VB_12_ 0.15 μg, PI metal solution 10 mL and chelated iron solution 3 mL; pH was 8.08.

PI metal solution (per L) comprised H_3_BO_3_ 3.426 g, CoCl_2_·6H_2_O 1.215 mg, MnCl_2_·4H_2_O 0.432 mg, ZnCl_2_ 31.5 mg, H_2_SO_4_ 1 mL and (NH_4_)_6_Mo_7_O_24_·4H_2_O 31.19 mg; chelated iron solution (per L) comprised Na_2_EDTA 10 g and FeCl_3_·6H_2_O 0.81 g.

### 3.2. Culture Conditions

Before inoculation, the microalgal seeds were observed under a microscope to check for bacterial contamination. The diatom *N. laevis* was cultured in 500 mL Erlenmeyer flasks containing 200 mL of fresh LDM medium, under dark condition with orbital shaking at 150 rpm at 22 °C. *P. tricornutum* was cultured in a photobioreactor containing 200 mL fresh f/2 medium. Light intensity was set at 50 μmol photons/m^2^/s and temperature was maintained at 22 °C. *I. galbana* was cultured in 500 mL Erlenmeyer flasks containing 200 mL of fresh f/2 medium and maintained in a stationary incubator under a 12/12 light/dark cycle, with the flasks manually shaken three times daily. All the culture mediums were supplemented with 5 g/L glucose.

### 3.3. Determination of Microalgal Biomass

Cell suspension (5 mL) was centrifuged at 5000 rpm for 3 min. Then, the pellet was washed twice with distilled water and filtered through a pre-weighed filter paper (Whatman GF/C). Cells on the filter paper were dried to a constant weight at 80 °C in a vacuum oven. The specific growth rates of *N. laevis*, *P. tricornutum* and *I. galbana* were calculated using the following equation:

(1) $$\mu =\left(\ln N_{t}-\ln N_{0}\right)/t$$

where $N_{t}$ was the biomass concentration of the culture after t days (g/L) and $N_{0}$ was the initial biomass concentration (g/L).

### 3.4. Analysis of Protein and Amino Acids

For the determination of intracellular protein, 10 mg lyophilized cells were added to 200 μL of 1 M NaOH, and heated in water bath at 80 °C for 10 min. Then, the samples were added with 1.8 mL distilled water and mixed well, centrifuged at 12,000× *g* for 30 min, and then, the supernatants were transferred to new centrifuge tubes. The extraction procedure was repeated twice, and all supernatants were collected to measure protein concentration by Protein assay kit (Bio-Rad, Hercules, CA, USA).

For the analysis of amino acids, 20 mg lyophilized cells were added to 6 M HCl and digested at 110 °C for 21 h. Then, 4 M NaOH solution was added to 50 μL digestion solution. Next, 50 μL protein precipitant was added to 50 μL mixed solution of the standards and samples, and then, 8 μL supernatant was mixed with 42 μL labeling buffer. The separated sample was mixed with 20 μL 6-Aminoquinolyl-N-hydroxysccinimidyl carbamate (AQC) as derivatization reagent, and incubated at 55 °C for 15 min. After derivatization, the samples were cooled in a refrigerator. To analyze the compositions and contents of amino acids, high-performance liquid chromatography (Ultimate 3000, Thermo Scientific, Waltham, MA, USA)-tandem mass spectrometry (API 3200 Q-TRAP, Applied Biosystems Inc., Foster City, CA, USA) (HPLC-MS/MS) was used [59].

The nutritional quality of protein from the microalgae was evaluated on the basis of *EAAI* [60]. The *EAAI* correlated the content of each EAA of a protein and the content of the same amino acid in the FAO/WHO standard (2013). The *EAAI* of microalgae examined here was calculated using the following equation:

(2) $$EAAI(\%)=\sqrt[{n}]{\left(\frac{U_{1}}{V_{1}}\right)\times \left(\frac{U_{2}}{V_{2}}\right)...\left(\frac{U_{n}}{V_{n}}\right)}$$

where *U* was the content of EAA (g/100 g), *V* was the content of the same amino acid in the FAO/WHO standard (g/100 g) and *n* was the number of analyzed EAAs.

### 3.5. Analysis of Carbohydrates and Monosac Charides

For the determination of total carbohydrates, 10 mg lyophilized cells were incubated with glacial acetic acid (0.1 mL) at 80 °C for 20 min. Then, 2 mL acetone was added followed by centrifugation at 5000 rpm for 10 min. Then, it was digested using 4 M trifluoroacetic acid by incubating in boiling water for 4 h. The suspension was cooled to room temperature and the supernatant was used to determine total sugar content by phenol sulphuric acid method [61]. To quantify the starch content, glucose was used to establish the standard curve.

For the analysis of monosaccharides, 5 mg lyophilized cells were added with 1 mL 2 M trifluoroacetic acid solution, then hydrolyzed in an oven at 110 °C for 6 h. After cooling to room temperature, the volume of sample solution was adjusted to 10 mL. Methanol solution was added to 2 mL sample solution and blown dry with nitrogen gas at 70 °C water bath. This step was repeated twice to remove trifluoroacetic acid. To obtain the polysaccharide hydrolysate, 1 mL 0.3 M NaOH solution was added to dissolve the residue. Next, 400 μL polysaccharide hydrolysate solution was added with 400 μL PMP methanol solution and incubated at 70 °C water bath for 2 h. When the solution was cooled to room temperature, 400 μL 0.3 M HCl (pH 6–7), 200 μL water, and 200 μL chloroform were added to the solution for extraction. High performance liquid chromatography (HPLC) was used to analyze the compositions and contents of monosaccharides [62,63].

### 3.6. Analysis of Lipids and Fatty Acids

For the determination of lipid content, 20 mg lyophilized cells were used with a mixture of chloroform, methanol and water (4:2:1.5, by volume). The chloroform layer was washed with 5% (*w*/*v*) sodium chloride and then evaporated under nitrogen gas; after that, it was dried at 60 °C in a vacuum oven to a constant weight and the samples were weighed. This extraction process was repeated multiple times until the weight before and after extraction remained constant to ensure complete extraction. The lipid content was determined based on the results from these multiple measurements. Chloroform was added to test tubes to adjust the final concentration at 10 μg chloroform/mg biomass [64].

For the analysis of fatty acids, 10 μL of total lipids was added to 2 mL 1% methanolic sulfuric acid solution (*v*/*v*, with 0.05% BHT) and 0.2 mL heptadecanoic acid methyl ester (HAME) solution (0.1 mg/mL n-hexane, *w*/*v*). The mixed solution was heated at 85 °C for 2.5 h with shaking every 30 min. The chloroform layer was taken to be methylated to fatty acid methyl esters (FAMEs) by incubating with 1% sulphuric acid in methanol [65]. After cooling to room temperature, the mixed solution was added to 1 mL 0.75% NaCl aqueous solution, and then extracted with 2 mL hexane. The FAMEs were analyzed by a gas chromatography-mass spectrometry (GC-MS, GC-MS-QP 2010 SE, Shimadzu, Japan), and quantified by a Stabliwas-DA capillary column (30 m × 0.25 mm × 0.25 μm, Shimadzu, Japan). A standard mixture of 37 FAMEs (C4:0-C22:6, Supleco Inc.) dissolved in n-hexane (30 mg/mL) was used to identify the methylated fatty acid species. HAME dissolved with n-hexane was used as the internal standard to quantify the contents of fatty acids. The injector temperature was 250 °C and injection volume was 1 μL.

### 3.7. Analysis of Pigments and Fucoxanthin

For the determination of pigment content, 20 mg lyophilized cells were dissolved in 99.9% methanol and incubated at 45 °C in the dark condition for 24 h. The mixture was centrifuged, and the absorbance of the supernatant was measured separately at 470, 652.4 and 665.2 nm. Pigment concentrations were calculated according to the previous study [66].

Fucoxanthin was extracted and the content was determined by HPLC according to our previous study [64]. The lyophilized cell samples were grounded and subsequently extracted with pure ethanol until the pellet was almost colorless. The ethanol layer was filtered through a 0.22-µm Millipore membrane before subjecting to HPLC.

### 3.8. Statistical Analysis

To ensure the accuracy of experimental analysis, the data of all the measurements were obtained from three repetitions and expressed as mean ± standard deviation (SD). One-way analysis of variance (ANOVA) with subsequent post hoc multiple-comparison LSD tests were used for significance analysis with SPSS 19.0 software.

## 4. Conclusions

This study analyzed and compared the cell growth and product content of *P. tricornutum*, *I. galbana* and *N. laevis* to assess the marine microalgae in the industrial applications. It was indicated that all the three microalgae possessed high nutritional value and promised to be natural sources of EAAs, fucoxanthin and PUFAs. *P. tricornutum* showed significantly higher levels of proteins and amino acids than the other two microalgae, while *I. galbana* had the highest total content of carbohydrates and lipids. *P. tricornutum* and *I. galbana* had higher fucoxanthin content compared to *N. laevis*, while among the three microalgae, *N. laevis* achieved the highest fucoxanthin and PUFAs productivities because of the competitive high cell density and the advantageous specific growth rate. Thus, among *P. tricornutum*, *I. galbana* and *N. laevis*, *N. laevis* was a more promising candidate for co-production of fucoxanthin and PUFAs at the industrial scale.

## Figures and Tables

**Table 1:** The carbon partitioning of *P. tricornutum*, *I. galbana* and *N. laevis* ^a^.

Carbon Partitioning Content (%DCW) ^b^	*P. tricornutum*	*I. galbana*	*N. laevis*
Protein	43.29 ± 0.76	31.60 ± 0.15	32.28 ± 0.25
Carbohydrate	14.85 ± 1.12	25.40 ± 1.38	21.97 ± 0.84
Lipid	23.56 ± 0.61	26.84 ± 0.70	21.25 ± 0.53
Pigment	7.31 ± 0.43	3.07 ± 0.22	2.45 ± 0.17

^a^ Data are expressed as mean ± SD of three replicates. ^b^ DCW: dry cell weight.

**Table 2:** Amino acid composition of *P. tricornutum*, *I. galbana* and *N. laevis* ^a^.

Amino Acid ^b^	FAO/WHO Standard (2013)	Amino Acid Content (mg/g)		
		*P. tricornutum*	*I. galbana*	*N. laevis*
Non-essential Amino Acid (NEAA)				
Gly		2.89 ± 011	3.32 ±0.04	2.20 ± 0.15
Ala		4.62 ± 0.09	6.39 ±0.06	3.50 ± 0.18
Ser		3.16 ± 0.08	3.83 ±0.01	3.19 ± 0.24
Cys		0.53 ± 0.01	0.70 ±0.01	0.53 ± 0.01
Asp		7.90 ± 0.23	9.11 ±0.09	6.38 ± 0.31
Asn		0.05 ± 0.00	0.06 ±0.00	0.03 ± 0.00
Glu		8.48 ± 0.23	12.37 ± 0.01	7.78 ± 0.41
Gln		0.49 ± 0.03	0.16 ± 0.00	0.09 ± 0.02
Tyr		0.83 ± 0.04	1.36 ± 0.08	0.87 ± 0.01
Pro		2.75 ± 0.04	3.61 ± 0.03	1.95 ± 0.10
Arg		4.30 ± 0.08	4.75 ± 0.16	4.21 ± 0.23
Essential amino acid (EAA)				
Val	4.00	3.51 ± 0.03	4.68 ± 0.07	2.85 ± 0.13
Thr	2.50	3.30 ± 0.12	4.11 ± 0.04	2.73 ± 0.17
Ile	3.00	3.33 ± 0.10	3.70 ± 0.08	2.23 ± 0.14
Met		1.02 ± 0.02	1.46 ± 0.05	0.74 ± 0.06
His	1.60	1.22 ± 0.01	1.68 ± 0.01	0.90 ± 0.06
Phe		3.47 ± 0.06	3.80 ± 0.01	2.01 ± 0.11
Trp	0.66		0.03 ± 0.00	
Lys	4.80	2.73 ± 0.17	2.82 ± 0.03	2.35 ± 0.08
Leu	6.10	5.57 ± 0.06	7.28 ± 0.03	4.03 ± 0.13
Phe + Tyr	4.10	4.30 ± 0.10	5.16 ± 0.09	2.88 ± 0.12
Met + Cys	2.3	1.55 ± 0.03	2.15 ± 0.06	1.27 ± 0.07
EAA/TAA		40.15 ± 1.06	39.29 ± 0.89	36.73 ± 2.99
EAA/NEAA		67.07 ± 2.33	64.73 ± 1.67	58.06 ± 4.14
EAAI		88.00 ± 4.67	77.30 ± 0.66	67.03 ± 6.17

^a^ Data are expressed as mean ± SD of three replicates. ^b^ Abbreviations: Isoleucine (Ile), leucine (Leu), valine (Val), lysine (Lis), phenylalanine (Phe), tyrosine (Tyr), methionine (Met), cysteine (Cys), tryptophan (Trp), threonine (Thr), alanine (Ala), arginine (Arg), aspartic acid (Asp), glutamic acid (Glu), glycine (Gly), histidine (His), proline (Pro), serine (Ser).

**Table 3:** Monosaccharide profiles of *P. tricornutum*, *I. galbana* and *N. laevis* ^a^.

Sugar	Abbreviation	Monosaccharide Content (mg/g)		
		*P. tricornutum*	*I. galbana*	*N. laevis*
Guluronic acid	GulA	10.89 ± 0.81	6.45 ± 0.78	2.27 ± 0.01
Mannuronic acid	ManA	0.72 ± 0.02		0.73 ± 0.03
Mannose	Man	41.16 ± 3.02	5.25 ± 0.45	44.26 ± 4.80
Ribose	Rib	2.96 ± 0.33	3.45 ± 0.34	4.25 ± 0.37
Rhamnose	Rham	3.49 ± 0.23	0.35 ± 0.02	1.33 ± 0.06
Glucosamine	GlcN	1.25 ± 0.08	0.63 ± 0.05	2.25 ± 0.04
Glucuronic acid	GlcUA	15.44 ± 0.55	4.79 ± 0.15	4.84 ± 0.13
Galacturonic acid	GalUA	1.00 ± 0.06	1.16 ± 0.08	2.85 ± 0.17
Galactosamine	GalN		0.21 ± 0.09	
Glucose	Glc	257.58 ± 13.30	38.11 ± 1.16	529.10 ± 2.55
Galactose	Gal	28.70 ± 1.22	23.98 ± 2.38	10.94 ± 0.95
Xylose	Xyl	6.46 ± 0.55	3.31 ± 0.18	3.60 ± 0.23
Arabinose	Ara	0.75 ± 0.05	7.01 ± 0.67	1.21 ± 0.11
Fucose	Fuc	1.72 ± 0.17	3.39 ± 0.29	2.24 ± 0.28

^a^ Data are expressed as mean ± SD of three replicates.

**Table 4:** Fatty acids profiles of *P. tricornutum*, *I. galbana* and *N. laevis* ^a^.

Fatty Acid	Fatty Acid Content (%DCW)		
	*P. tricornutum*	*I. galbana*	*N. laevis*
C14:0	0.98 ± 0.00	1.71 ± 0.01	0.83 ± 0.10
C16:0	1.57 ± 0.01	1.82 ± 0.02	1.36 ± 0.12
C16:1	3.41 ± 0.01	0.32 ± 0.01	2.89 ± 0.12
C16:2	0.32 ± 0.01		0.45 ± 0.00
C16:3	0.50 ± 0.01		
C18:1	0.65 ± 0.00	3.17 ± 0.03	0.19 ± 0.00
C18:2*n*-6	0.27 ± 0.01	0.29 ± 0.00	0.19 ± 0.00
C18:3*n*-3/6		1.05 ± 0.01	0.30 ± 0.01
C18:4*n*-3		1.20 ± 0.03	0.13 ± 0.01
C20:4*n*-6		0.22 ± 0.00	0.33 ± 0.02
C20:5*n*-3	2.17 ± 0.03		2.18 ± 0.14
C22:6*n*-3		0.91 ± 0.01	0.18 ± 0.00
others	0.22 ± 0.00	0.28 ± 0.02	0.65 ± 0.01
TFA	10.09 ± 0.06	10.95 ± 0.02	9.66 ± 0.22

^a^ Data are expressed as mean ± SD of three replicates.

## Data Availability

The data presented in this study are available on request from the corresponding author.
